# Group I metabotropic glutamate receptor mediated dynamic immune dysfunction in children with fragile X syndrome

**DOI:** 10.1186/1742-2094-11-110

**Published:** 2014-06-19

**Authors:** Milo Careaga, Tamanna Noyon, Kirin Basuta, Judy Van de Water, Flora Tassone, Randi J Hagerman, Paul Ashwood

**Affiliations:** 1 Department of Medical Microbiology and Immunology, and the MIND Institute, UC Davis, 2805, 50th Street, Sacramento, CA 95817, USA; 2 MIND Institute, UC Davis Medical Center, Sacramento, CA, USA; 3 Department of Biochemistry and Molecular Medicine, UC Davis, Davis, CA, USA; 4 Division of Rheumatology, Allergy and Clinical Immunology, UC Davis, Davis, CA, USA; 5 Department of Pediatrics, University of California at Davis, Davis, CA, USA

**Keywords:** Fragile x syndrome, Immune, Cytokine, Metabotropic glutamate receptor

## Abstract

**Background:**

Fragile X syndrome (FXS) is the leading cause of inheritable intellectual disability in male children, and is predominantly caused by a single gene mutation resulting in expanded trinucleotide CGG-repeats within the 5’ untranslated region of the fragile X mental retardation (*FMR1*) gene. Reports have suggested the presence of immune dysregulation in FXS with evidence of altered plasma cytokine levels; however, no studies have directly assessed functional cellular immune responses in children with FXS. In order to ascertain if immune dysregulation is present in children with FXS, dynamic cellular responses to immune stimulation were examined.

**Methods:**

Peripheral blood mononuclear cells (PBMC) were from male children with FXS (n = 27) and from male aged-matched typically developing (TD) controls (n = 8). PBMC were cultured for 48 hours in media alone or with lipopolysaccharides (LPS; 1 μg/mL) to stimulate the innate immune response or with phytohemagglutinin (PHA; 8 μg/mL) to stimulate the adaptive T-cell response. Additionally, the group I mGluR agonist, DHPG, was added to cultures to ascertain the role of mGluR signaling in the immune response in subject with FXS. Supernatants were harvested and cytokine levels were assessed using Luminex multiplexing technology.

**Results:**

Children with FXS displayed similar innate immune response following challenge with LPS alone when compared with TD controls; however, when LPS was added in the presence of a group I mGluR agonist, DHPG, increased immune response were observed in children with FXS for a number of pro-inflammatory cytokines including IL-6 (*P* = 0.02), and IL-12p40 (*P* < 0.01). Following PHA stimulation, with or without DHPG, no significant differences between subjects with FXS and TD were seen.

**Conclusions:**

In unstimulated cultures, subjects with FXS did not display altered dynamic immune response to LPS or PHA alone; however, subjects with FXS showed an altered response to co-current stimulation of LPS and DHPG, such that subjects with FXS failed to inhibit production of pro-inflammatory cytokines, suggesting a role of group I mGluR signaling in innate immune responses in FXS.

## Introduction

Fragile X syndrome (FXS) is a single-gene disorder nearly always caused by an unstable mutation in the fragile X mental retardation 1 (*FMR1*) X-linked gene and results from the expansion of a trinucleotide (CGG) repeat sequence in the 5’UTR of this gene [1]. The full mutation, present in individuals having an *FMR1* allele with more than 200 CGG repeats, typically is methylated with partial or complete transcriptional silencing of the *FMR1* gene, leading to a reduction or absence of the *FMR1* protein, FMRP [1-4]. Although FXS is associated with a characteristic phenotype, there is considerable within-syndrome variation in the severity of affectedness and the profile of impairments including a significant (approximately 30%) comorbidity with autism spectrum disorders (ASD) [5,6].

Recent evidence suggests that there is immune dysregulation in individuals with FXS that may play a role in the disease process [7]. Neuroimmune interactions begin during early neurodevelopment and continue throughout life, with the immune system supporting many aspects of neural function. Alterations in the immune system would increase sensitivity to neurologic damage from a variety of sources including infection and exposure to xenobiotics, particularly during early development. Indeed, an increased frequency of infections has been reported in a subgroup of boys with FXS, especially in early childhood [8]. This may be the result of an aberrant or dysfunctional immune response in this group, and possibly contributes to the frequently observed severe cognitive deficit and language impairment [8]. Consequently, there is a need for research that can identify the biological basis of the immune anomalies in FXS, which may facilitate future treatments.

Groundbreaking advances in the FXS field have paved the way for treatment of the underlying neurobiology of the disorder. The ‘mGluR theory’ of FXS, which is strongly supported by several lines of evidence, states that mental impairment and phenotypic behaviors associated with FXS arise, at least in part, from constitutive activation of translational pathways normally controlled by group 1 metabotropic glutamate receptor (mGluR1 and mGluR5) activity [9]. Group I mGluRs are involved in numerous functions including learning and memory, and are expressed in both the central and peripheral nervous system [10]. In neurons, mGluRs can augment synapse excitability and thereby alter the function of other receptors including GABA receptors [11].

mGluRs are expressed on peripheral blood mononuclear cells (PBMC), where they are believed to be involved in immune development, activation, response, and survival [12-15]. mGluRs are members of the group C family of G-protein-coupled receptors, and function by signaling through G proteins to activate secondary messengers. They are responsive to low concentrations of glutamate, such as that observed in the periphery, where glutamate levels are typically between 20 to 40 μM in plasma [16]; considerably lower than what is found in synaptic clefs within the central nervous system (CNS) where glutamate levels can reach millimolar levels [17]. Group I mGluRs in particular, are expressed differentially in naïve and activated T-cells, with mGluR5 being constitutively expressed and mGluR1 only being expressed in activated T-cells [18]. In addition, mGluRs on T-cells have been shown to be differentially coupled to intracellular signaling systems such as cAMP, and ERK pathways [15]. It is thought that the differential expression on the cell surface and coupling to signaling mechanisms contributes to T-cell activation, expression of pro-inflammatory cytokines, and cell survival. In addition, mGluRs are also differentially expressed on other immune cells such as microglia and macrophages and may be related to activation status [19].

The present study focused on how group I mGluR activation influences the immune response in pediatric populations of typically developing (TD) children compared to children with FXS, through an analysis of dynamic immune cell function. To date, no study has attempted to analyze the function of neurotransmitter receptors using PBMC in FXS. This study illustrates the role of mGluRs in immune cell function in both a neurotypical pediatric population as well as in children with FXS where mGluR signaling is altered, expanding upon previous immunological findings in FXS.

## Material and methods

### Subjects

Twenty-seven male subjects with FXS aged 2 to 9 years (median 5.4 years (interquartile range 3.7 to 7.5)) were recruited through the Fragile X Treatment and Research Center at the MIND Institute at University of California, Davis, CA, USA. The study also included 8 male TD controls aged 3 to 8 years (median 3.3 years (3.2 to 5.7 years)). Subjects on minocycline or other medications with established anti-inflammatory properties were excluded from the study. All participants with FXS underwent a clinical assessment that included a detailed medical history and medical examination, and measurements of typical genetic and physical features of FXS. In addition, subjects underwent IQ testing and adaptive skills testing using the following instruments: the Wechsler Scales of Intelligence [20] and the Vineland Adaptive Behavior Scales [21]. Controls were administered a medical examination and the Social Communication Questionnaire (SCQ) [22] to determine that they were typically developing, and none exceeded scores above 15. In addition, a review of available prenatal, birth, and medical records were performed for all subjects. Clinical assessments were performed by trained clinicians under the supervision of one of the authors (RJH), a pediatrician at the MIND Institute. The study protocol followed the ethical guidelines of the declaration of Helsinki and was approved by the Institutional Review Boards of the UC Davis School of Medicine and the State of California, and written informed consent was obtained from a legal guardian for all study subjects.

### CGG sizing

Genomic DNA was isolated from 3 to 5 mL of peripheral blood leukocytes using standard procedures (Gentra Puregene kit; Qiagen, Valencia, CA). An initial centrifugation step on whole blood permitted plasma separation and storage before isolation of the DNA.

The size and methylation status of the CGG repeats was determined using both Southern blot and PCR analysis. Details of the Southern blot and PCR methods are in Tassone *et al*. [23] and Filipovic-Sadic *et al*. [24]. Analysis and calculation of the repeat size for both Southern blot and PCR analysis was carried out using an Alpha Innotech FluorChem 8800 Image Detection System (ProteinSimple, Santa Clara, CA)and the ABI 3730XL 96-Capillary Electrophoresis Genetic Analyzer (Life Technologies, Grand Island, NY).

### Cell isolation

Peripheral blood was collected in an acid-citrate-dextrose Vacutainer tube (BD Biosciences; San Jose, CA, USA) and processed within 12 hours of collection. Blood was centrifuged for 10 minutes at 2,300 rpm, and plasma was collected and stored at −80°C. The remaining cells were mixed 1:1 with Hanks Balanced Salt Solution (HBSS; Gibco, Gaithersburg, MD, USA) without Ca^2+^ or Mg^2+^. The diluted blood was then carefully layered over a Ficoll-Paque gradient (Pharmacia Biotech, Piscataway, NJ, USA) and centrifuged at 1,700 rpm for 30 minutes at room temperature. PBMC were then harvested from the interface layer and washed twice with HBSS. Viability was be determined by trypan blue exclusion. Cells were then resuspended at a concentration of 2 × 10^6^ cells/mL in tissue culture medium (TCM) consisting of: RPMI 1640 (Gibco, Gaithersburg, MD, USA) supplemented with 10% low endotoxin, heat inactivated FBS (Omega Scientific; Tarzana, CA, USA), 100 IU/mL penicillin, and 100 IU/mL streptomycin (Sigma, St Louis, MO, USA).

### Cellular stimulations

Isolated PBMC were stimulated for one hour in RPMI 1640 media with 10% FBS (Gibco, Gaithersburg, MD, USA), 1% penicillin and streptomycin alone, or the addition of 100 μM of a group I mGluR agonist (S)-3,5-dihydroxyphenylglycine (DHPG) (DHPG; Tocris, Minneapolis, MN, USA), or 10 μM of an mGluR5 specific antagonist 3-((2-methyl-1,3-thiazol-4-yl)ethynyl)pyridine hydrochloride (MTEP; Tocris, Minneapolis, MN, USA) followed by the addition of either 1.0 μg/mL lipopolysaccharide (LPS; *Escherichia coli* serotype 0111:B4, Sigma, St. Louis, MO, USA) or 8 μg/mL phytohemagglutinin (PHA; Sigma, St Louis, MO, USA) and cultured at 37°C with 5% CO_2_ for another 47 hours (48 hours total). After this period, cells were collected and spun at 2,000 rpm for 10 minutes and supernatants were collected and stored at −80°C until analyzed by Luminex multiplexing technology.

### Luminex multiplex analysis

Quantification of IFNγ, granulocyte-macrophage colony-stimulating factor (GM-CSF), IL-1β, IL-6, IL-10, IL-12(p40), IL-13, IL-17 and TNFα in the cell supernatants was determined using human multiplexing bead immunoassays (Millipore, Billerica, MA, USA). The cytokines GM-CSF, IL-1β, IL-6, IL-10, TNFα, and IL-12(p40) were used to evaluate innate immune responses after LPS stimulation and the cytokines GM-CSF, IFNγ, IL-10, IL-13, and IL-17 were used to assess responses after PHA stimulation. Samples were analyzed per manufacturer specifications. Specifically, 25 μL of supernatant were incubated with antibody-coupled beads. After a series of washes, a biotinylated detection antibody was added to the beads, and the reaction mixture was detected by the addition of streptavidin-phycoerythrin. The bead sets were analyzed using a flow-based Luminex™ 100 suspension array system (Bio-Plex 200; Bio-Rad Laboratories, Inc. Hercules, CA, USA). Unknown sample cytokine concentrations were calculated by Bio-Plex Manager software (Bio-Rad Laboratories, Inc. Hercules, CA, USA) using a standard curve derived from the known reference cytokine concentrations supplied by the manufacturer. A five-parameter model was used to calculate final concentrations and values are expressed in pg/mL. The sensitivity of this assay allowed the detection of cytokine concentrations with the following limits of detection: IFNγ (0.4 pg/mL), GM-CSF (2.3 pg/mL), IL-1β (0.7 pg/mL), IL-6 (0.4 pg/mL), IL-10 (0.3 pg/mL), IL-12(p40) (12.3 pg/mL), IL-13 (0.3 pg/mL), IL-17 (0.4 pg/mL), and TNFα (0.2 pg/mL). Values below the limit of detection (LOD) were replaced with one half the LOD. Supernatant aliquots were free of any previous freeze/thaw cycle.

### Statistical analysis

Data analysis was performed using STATA 12 software (College Station, TX, USA). Data was determined as non-parametric using Shapiro-Wilks test for normality. Wilcoxon matched-pairs signed-rank tests were used to compare cytokine levels within group pre and post stimulation and Wilcoxon-rank sum tests for between subject group comparisons. For comparison of relative immune response between group, outliers were determined if datum were greater than four median absolute deviations from the mean. A probability value (*P*) of less than 0.05 was considered to be significant.

## Results

PBMC from children with FXS and TD controls were stimulated with LPS, a Toll-like receptor (TLR)-4 agonist, for 48 hours to assess the dynamic response of their innate immune system. No significant differences were apparent in the supernatants collected from the cell cultures of TD controls compared with children with FXS in the cytokines assayed (GM-CSF, IL-1β, IL-6, IL-10, TNFα, and IL-12(p40)) (Table 1). When a group I mGluR agonist, DHPG, was added during stimulation there was a significant decrease in cytokines levels for GM-CSF (*P* = 0.04), IL-12(p40) (*P* = 0.01), and TNFα (*P* = 0.03) in TD controls (Table 1). In contrast, cell culture supernatants from children with FXS showed that select inflammatory cytokines levels were increased or remained unchanged after administration of DHPG to the culture (*P* < 0.01; Table 1). Further, the anti-inflammatory cytokine, IL-10, was decreased following stimulation in the presence of DHPG (*P* < 0.01; Table 1). The exception to these findings was IL-1β production, which was marginally increased in TD controls (*P* = 0.05) but still had a greater production in FXS children, with an over two-fold increase (*P* < 0.01) when PBMC were stimulated with LPS plus DHPG (Table 1).

**Table 1:** Cytokine levels in lipopolysaccharide (LPS)-stimulated and LPS plus DHPG-stimulated cell cultures

	**TD**			**FXS**		
**Cytokine**	**LPS**	**LPS + DHPG**	***P*-value**	**LPS**	**LPS + DHPG**	***P*-value**
GM-CSF	50.4 (27.9 to 58.9)	39.6 (19.5 to 42.9)	0.04^a^	45.2 (17.7 to 70.7)	55.3 (20.1 to 107.2)	0.28
IL-1β	1,245.3 (957.4 to 1,855.5)	1,567.6 (1,160.2 to 2,235.0)	0.05^a^	1,146.0 (607.9 to 1,748.2)	2,326.9 (952.6 to 3,313.6)	< 0.01^b^
IL-6	3,203.9 (2,043.8 to 5,14.0)	2,856.1 (1,701.6 to 4,715.8)	0.33	2,900.2 (1,792.2 to 5,110.3)	4,366.9 (2,047.0 to 6,251.1)	0.21
IL-10	271.2 (106.3 to 765.6)	100.0 (85.0 to 515.8)	0.40	286.8 (184.0 to 573.0)	179.3 (76.3 to 342.1)	0.01^b^
IL-12(p40)	59.2 (36.9 to 72.5)	24.1 (16.3 to 51.2)	0.01^a^	25.0 (6.2 to 77.3)	46.1 (15.1 to 91.0)	0.37
TNFα	838.4 (626.3 to 1,034.4)	570.7 (485.1 to 914.5)	0.03^a^	724.2 (413.0 to 1,381.2)	1,010.4 (458.1 to 1,510.4)	0.49

Values reported as median (interquartile range) in pg/mL. All *P*-values were calculated by Wilcoxon matched-pair signed-rank test. ^a^*P* < 0.05; ^b^*P* < 0.01. DHPG, (S)-3,5-dihydroxyphenylglycine; FXS, fragile X syndrome; TD, typically developing.

**Figure 1:**
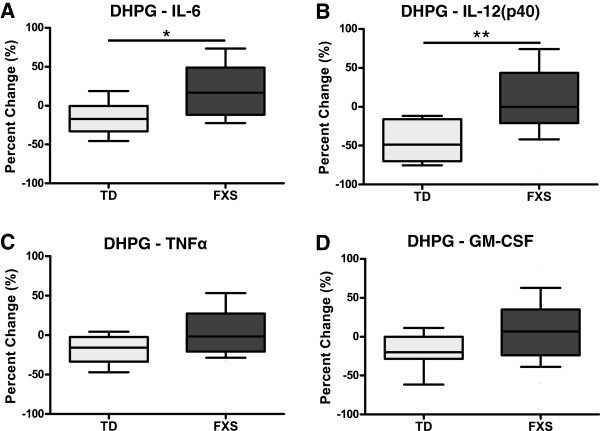
**Peripheral blood mononuclear cells’ (PBMC) response to lipopolysaccharide (LPS) stimulation with a group I mGluR agonist.** PBMC from children with fragile X syndrome (FXS) displayed an opposite or exaggerated immune response to LPS and (S)-3,5-dihydroxyphenylglycine (DHPG) relative to LPS alone when compared with typically developing (TD) controls for **(A)** IL-6 (*P* = 0.02), **(B)** IL-12(p40) (*P* < 0.01), and similar albeit non-significant trends for both **(C)** TNFα (*P* = 0.07) and **(D)** GM-CSF (*P* = 0.11). *P < 0.05, **P < 0.01.

Overall, the apparent skewing in cytokine production could be observed as a differential response to LPS stimulation in the presence of DHPG compared with LPS alone (Figure 1A-D); such that TD controls displayed a significantly lower production of IL-6 (median −17.3% (interquartile range −33.0 to −0.4%) versus 11.4% (−11.7 to 43.6%); *P* = 0.02) and IL-12(p40) (median −48.7% (interquartile range −69.7 to −19.8%) versus 0% (−21.1 to 43.4%); *P* < 0.01) in the presence of DHPG compared with children with FXS. In addition, both TNFα (median −15.5% (interquartile range −30.8 to −4.8%) versus −1.6% (−18.3 to 25.8); *P* = 0.07) and GM-CSF (median −19.9% (interquartile range −28.0 to −1.7%) versus 6.7% (−23.5 to 33.8%); *P* = 0.11) showed trends toward a higher differential response in TD controls compared children with FXS approaching statistical significance.

**Figure 2:**
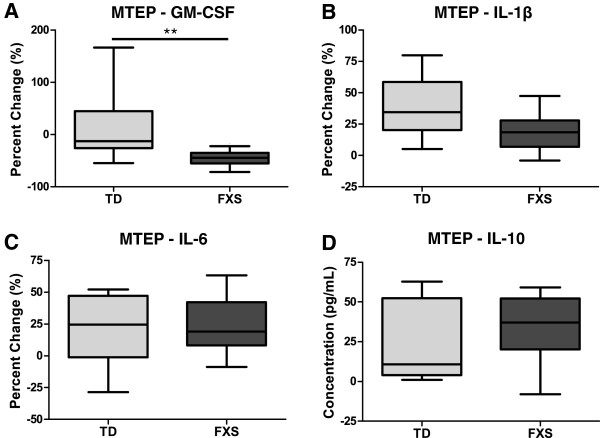
**Peripheral blood mononuclear cells’ (PBMC) response to lipopolysaccharide (LPS) stimulation with a mGluR5 antagonist.** PBMC from children with fragile X syndrome (FXS) displayed an opposite immune response to LPS and 3-((2-methyl-1,3-thiazol-4-yl)ethynyl)pyridine hydrochloride (MTEP) relative to LPS alone when compared with typically developing (TD) controls for **(A)** GM-CSF (*P* < 0.01). **(B)** IL-1β, **(C)** IL-6, and **(D)** IL-10 showed similar responses to MTEP in both subjects and controls. *P < 0.05, **P < 0.01.

To determine if the basal level of glutamate in the culture media was altering baseline response of PBMC via a group I mGluR-dependent manner in children with FXS, a group I mGluR antagonist, MTEP, was added to the culture during LPS stimulation (Figure 2A-D). In TD controls, cytokine responses tended to be higher with significantly increased production of both IL-1β (*P* = 0.03) and IL-10 (*P* = 0.04) detected (Table 2), as anticipated, largely having the opposite trend as was seen with the group mGluR agonist DHPG. Children with FXS also tended to show increased responses, with elevated levels of IL-1β (*P* < 0.01), IL-6 (*P* < 0.01), and IL-10 (*P* < 0.01). However, the production of GM-CSF was significantly decreased (*P* < 0.01) under these conditions (Table 2). When the relative response to LPS and MTEP to LPS alone was compared between TD controls and children with FXS, GM-CSF ((−16.1% (−16.5 to 8.7%) versus −44.3% (−54.4 to −35.6%); *P* = 0.02) responses were reduced significantly more in children with FXS.

**Table 2:** Cytokine levels in lipopolysaccharide (LPS)-stimulated and LPS plus MTEP-stimulated cell cultures

	**TD**			**FXS**		
**Cytokine**	**LPS**	**LPS + MTEP**	***P*-value**	**LPS**	**LPS + MTEP**	***P*-value**
GM-CSF	50.4 (27.9 to 58.9)	39.8 (36.5 to 49.4)	0.35	45.2 (17.7 to 70.7)	25.5 (11.7 to 57.6)	< 0.01^b^
IL-1β	1,245.3 (957.4 to 1,855.5)	1,545.9 (972.5 to 2,312.1)	0.03^a^	1,146.0 (607.9 to 1,748.2)	1,558.4 (883.3 to 2,148.6)	< 0.01^b^
IL-6	3,203.9 (2,043.8 to 4,514.0)	4,195.3 (2,235.9 to 6,071.8)	0.12	2,900.2 (1,792.2 to 5,110.3)	3,969.3 (2,352.4 to 7,203.9)	0.01^a^
IL-10	271.2 (106.3 to 765.6)	415.6 (168.7 to 1,076.6)	0.03^a^	286.8 (184.0 to 573.0)	389.2 (210.4 to 608.2)	< 0.01^b^
IL-12(p40)	59.2 (36.9 to 72.5)	89.8 (17.3 to 101.8)	0.53	25.0 (6.2 to 77.3)	43.7 (22.5 to 96.3)	0.44
TNFα	838.4 (626.3 to 1,034.4)	922.5 (531.7 to 1,190.7)	0.75	724.2 (413.0 to 1,381.2)	925.1 (516.7 to 1,557.1)	0.93

Values reported as median (interquartile range) in pg/mL. All *P*-values were calculated by Wilcoxon matched-pair signed-rank test. ^a^*P* < 0.05; ^b^*P* < 0.01. FXS, fragile X syndrome; MTEP, 3-((2-methyl-1,3-thiazol-4-yl)ethynyl)pyridine hydrochloride; TD, typically developing.

The adaptive immune responses were compared between TD controls and children with FXS by measuring cytokine production after culturing PBMC with a cellular mitogen, PHA, which preferentially activates T-cells. No significance differences were apparent between the two groups (Table 3). Further, the addition of DHPG to the PHA cultures did not alter the cytokine production in TD controls. However, a significant decrease in T_H_2 associated cytokines IL-10 (*P* < 0.01) and IL-13 (*P* = 0.01) was observed in children with FXS versus the TD controls (Table 3). Unlike the innate response, no significant differential response between PHA and DHPG relative to PHA alone was apparent between groups (data not shown). The addition of MTEP to the PHA cultures resulted in a moderate but not significant increase in IL-10 production (*P* > 0.05) in TD controls. In children with FXS, there were decreases in cytokine production with significantly lower levels of GM-CSF (*P* < 0.01), and lower (not significant) levels of IL-13 (*P* = 0.12) (Table 4).

**Table 3:** Cytokine levels in phytohemagglutinin (PHA)-stimulated and PHA plus DHPG-stimulated cell cultures

	**TD**			**FXS**		
**Cytokine**	**PHA**	**PHA + DHPG**	***P*-value**	**PHA**	**PHA + DHPG**	***P*-value**
GM-CSF	8.4 (2.3 to 76.9)	9.6 (4.6 to 45.1)	0.36	11.0 (4.2 to 32.7)	8.2 (5.2 to 25.8)	0.81
IFNγ	9.3 (5.3 to 122.1)	9.9 (5.1 to 113.0)	0.72	8.6 (3.0 to 113.2)	5.6 (1.3 to 221.9)	0.89
IL-10	40.3 (17.7 to 144.3)	34.2 (25.5 to 117.5)	1.00	44.0 (16.5 to 115.0)	26.9 (15.3 to 74.6)	< 0.01^b^
IL-13	6.2 (3.4 to 135.9)	7.4 (3.1 to 82.4)	0.09	6.1 (0.2 to 22.1)	2.6 (0.2 to 18.8)	< 0.01^b^
IL-17	15.4 (6.5 to 70.1)	15.4 (6.9 to 40.2)	0.73	18.0 (2.7 to 43.6)	12.1 (5.1 to 34.3)	0.18

Values reported as median (interquartile range) in pg/mL. All *P*-values were calculated by Wilcoxon matched-pair signed-rank test. ^b^*P* < 0.01. DHPG, (S)-3,5-dihydroxyphenylglycine; FXS, fragile X syndrome; TD, typically developing.

**Table 4:** Cytokine levels in phytohemagglutinin (PHA)-stimulated and PHA plus MTEP-stimulated cell cultures

	**TD**			**FXS**		
**Cytokine**	**PHA**	**PHA + MTEP**	***P-*** **value**	**PHA**	**PHA + MTEP**	***P*-value**
GM-CSF	8.4 (2.3 to 76.9)	3.7 (2.6 to 14.5)	0.83	11.0 (4.2 to 32.7)	3.5 (1.2 to 12.1)	0.01^a^
IFNγ	9.3 (5.3 to 122.1)	4.4 (1.4 to 14.4)	0.83	8.6 (3.0 to 113.2)	1.5 (1.3 to 8.6)	0.23
IL-10	40.3 (17.7 to 144.3)	34.0 (29.4 to 60.8)	0.05	44.0 (16.5 to 115.0)	40.4 (9.3 to 67.7)	0.46
IL-13	6.2 (3.4 to 135.9)	8.1 (2.7 to 29.1)	0.67	6.1 (0.2 to 22.1)	0.2 (0.2 to 9.6)	0.12
IL-17	15.4 (6.5 to 70.1)	12.6 (6.5 to 35.6)	0.25	18.0 (2.7 to 43.6)	5.9 (0.9 to 20.2)	0.69

Values reported as median (interquartile range) in pg/mL. All *P*-values were calculated by Wilcoxon matched-pair signed-rank test. ^a^*P* < 0.05. FXS, fragile X syndrome; MTEP, 3-((2-methyl-1,3-thiazol-4-yl)ethynyl)pyridine hydrochloride; TD, typically developing.

## Discussion

Systemic immune differences have previously been noted in children with FXS compared with TD controls [7]. This included increased IL-1α, as well as decreased RANTES and IP-10 in plasma. In addition, increased rates of infection have been observed in children with FXS, suggesting that immune dysregulation or non-optimal immune function may occur in these children leading to increased susceptibility to infection [8]. However, it is not clear if this immune dysfunction results from differences in the cellular immune response following immune challenge, or from other physiological factors. In this study we found that peripheral blood immune cells from children with FXS respond to LPS and PHA in a similar fashion as cells from TD children. This suggest that differences in plasma cytokine values previously observed could result from other physiological stresses observed in FXS, particularly increased anxiety, as anxiety levels have been shown to relate to inflammation in a number of studies [25]. Alternatively, immune differences might be restricted to specific anatomical compartments, such as the gastrointestinal tract or liver, which can affect circulating levels of cytokines while analysis of circulating immune cells may not adequately reflect immune differences at these sites.

Group I mGluRs have also been implicated in the pathogenesis of FXS [26]. In FXS, the decreased production of FMRP is thought to lead to a lack of dampening of group I mGluR signaling, resulting in aberrant activation of these receptors. Down-regulation of group I mGluRs in a mouse model of FXS has demonstrated that this is sufficient to ameliorate most of pathological effects seen in FXS [27], suggesting group I mGluRs are highly involved in the pathogenesis of FXS. Although it is difficult, if not impossible, to assess mGluR function in the CNS of children, their peripheral expression (including most immune cells) provides a novel opportunity to assess mGluR function in a non-invasive way. Utilizing this alternative source of mGluRs, we sought to determine if group I mGluR signaling could be assessed in subjects by taking advantage of the known ability of mGluRs to modulate the immune system [13,28]. Given the purported mGluR expression and signaling differences in FXS, we hypothesized that analysis of mGluR in the context of immune stimulation could help to explain immune differences previously seen in the periphery in subjects with FXS.

In the presence of the group I mGluR agonist, DHPG, activation of the innate immune system demonstrated that TD controls generally showed a decrease in cytokine production relative to LPS alone. This is similar to the response of microglia following administration of DHPG where a decrease the inflammatory response to LPS has been noted [29]. The activation of mGluR5 has also been shown to reduce microglial associated inflammation and neurotoxicity [30]. Thus, PBMC from TD controls respond to DHPG in a manner similar to central myeloid cells, that is decreased inflammatory cytokine response in the presence of immune challenge. However, immune cells from children with FXS when exposed to DHPG in the presence of LPS did not show the same level of inhibition of inflammation as the TD controls. In the CNS, glutamate serves many purposes, including acting as a messenger between neurons and microglia, and it has also been shown that glutamate signaling in microglia could serve a protective role [31]. Therefore, dysregulation of group I mGluR-mediated inhibition in microglia would have potentially devastating effects and lead to negative outcomes such as reduced immune regulation with an increase in neuroinflammation. Agents known to reduce neuroinflammation, such as the tetracycline derivative minocycline, have been shown to rescue many of the impairments seen in the *FMR1* knockout (KO) mouse when administered during early development [32]. The observed pattern of immune dysregulation following LPS coupled with DHPG in our study would also explain previous observations in the *FMR1* KO mouse. When LPS was given to *FMR1* KO mice, similar peripheral immune responses were seen in comparison to wild-type (WT) controls, with the KO mice demonstrating both normal peripheral immune responses and normal microglia responses in culture. However, isolates from the brains of these animals, where glutamate levels would be the highest, showed signs of immune activation and neuroinflammation [33].

The addition of DHPG to PHA-stimulated cell cultures resulted in similar levels of cytokine production between children with FXS and controls. In general, results were much more variable than in the LPS cultures. As PHA preferentially activates T-cells, this might relate to previous findings regarding the ability of mGluRs to regulate T-cell immunity. Not only are mGluR1 and mGluR5 differentially coupled to separate signaling systems in lymphocytes, their surface expression differs depending on the state of the cell. Immature T-cells show little or no mGluR1 expression unlike mature T-cells, and mGluR5 seems to be constitutively expressed by these cells [15,18]. As both the phosphatidylinositide 3-kinase (PI3K) and mitogen-activated protein kinase (MAPK) pathways have been suggested to be dysregulated in FXS [34,35], and as these pathways are involved in T-cell maturation and survival [36], altered mGluR expression may be masking the difference in mGluR function in T-cells.

Blocking of group I mGluR through pharmaceutical means has proven to be beneficial in animal models of FXS. In mouse models of FXS, administration of the group I mGluR antagonists, 2-methyl-6-(phenylethynyl)pyridine hydrochloride (MPEP), has been shown to reverse a number of phenotypes including autogenic seizures and abnormal open field exploration [37], deficits in prepulse inhibition [38], decreased mRNA granule expression [39], excess protein in hippocampal slices [40], and increased density of dendritic filopodia in hippocampal cultures [38]. In our study, administration of MTEP to the immune cell cultures stimulated with LPS resulted in lower production of GM-CSF in children with FXS compared with TD controls. Although MTEP is more specific than MPEP for the mGluRs, it has a short half-life and might not have been fully effective in suppressing all mGluR5 for the duration of the stimulation used herein [41]. Several newer inhibitors are in production and might better serve as agents to test the role mGluR antagonist on dynamic immune response.

**Figure 3:**
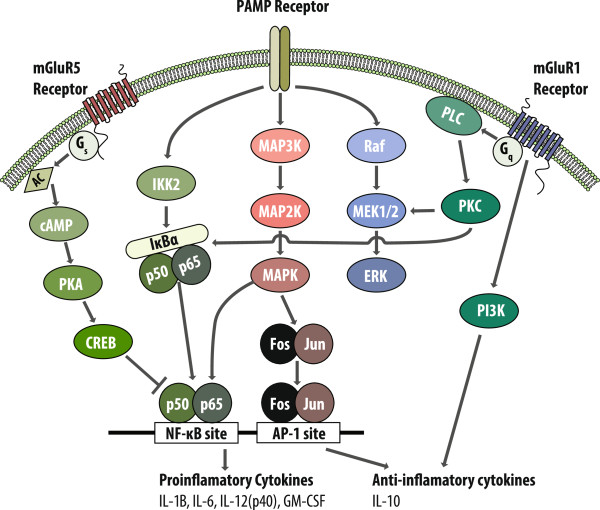
**Group I mGluR-signaling pathway in immune cells.** Activation of pathogen associated molecular pattern (PAMP) receptors leads to a signaling cascade which can be both inhibited and assisted by group I mGluR signaling.

Although it is not fully known how mGluRs regulate the immune response, many of the pathways mGluRs signal through are convergent with signaling pathways utilized by immune cell receptors. Immune cells sense LPS though TLR4, a pathogen-associated molecular pattern (PAMP) receptor, which leads to a signaling cascade and the activation of the nuclear factor kappa-light-chain-enhancer of activated B cells (NFκB) and the MAPK pathways. The activation of these pathways culminates in the activation of transcription factors, which in turn promote the production of pro-inflammatory cytokines as well as anti-inflammatory cytokines. Group I mGluRs can inhibit or assist these pathways. Although not fully characterized in myeloid cells, in lymphocytes mGluR1 is coupled to G_αq_ and activates the phospholipase C (PLC), PI3K, and MAPK pathways [18]. mGluR5, however, preferentially couples to G_αs_, which activates the adenylyl cyclase and leads to the production of the secondary messenger 3’,5’-cyclic AMP (cAMP) [18]. Whereas mGluR1 pathways tend to assist PAMP signaling, mGluR5 inhibits through the production of cAMP (Figure 3). In FXS, cAMP tends to be lower and cAMP metabolism has been shown to be dysregulated [42-44]. This dysregulation of the cAMP system might be hindering the effects of mGluR5 in myeloid cells in children with FXS, leading to a lack of inhibition of cytokine production when stimulated. This altered response to glutamate by mGluR could have dire effects of the ability of the immune system in the CNS to regulate immune homeostasis, and could have implications into the pathology of FXS.

## Conclusion

Children with FXS did not display an altered dynamic immune response to LPS at a cellular level; however, subjects with FXS showed an altered response to LPS with DHPG, leading to increased production of inflammatory cytokines. Stimulation of immune cells from subjects with FXS with PHA or PHA with DHPG resulted in no difference in cytokine production in immune cells from similarly treated TD controls. In addition, this study demonstrates that group I mGluR functionality can be tested using a surrogate cellular system instead of neurons. These findings should be valuable in the generation of testable hypothesis to understand how immune and mGluR activation can influence neuronal dysfunction and alter patterns of early brain development in FXS. In addition, mGluR function in PBMC could help to better establish efficacy of drug studies utilizing mGluR-modulating agents.

## Abbreviations

ASD: autism spectrum disorders; cAMP: 3’,5’-cyclic AMP; CNS: central nervous system; DHPG: (S)-3,5-dihydroxyphenylglycine; FBS: fetal bovine serum; FMR1: fragile X mental retardation 1; FMRP: fragile X mental retardation protein; GM-CSF: granulocyte-macrophage colony-stimulating factor; HBBS: Hanks balanced salt solution; IFN: interferon; IL: interleukin; KO: knockout; LOD: limit of detection; LPS: lipopolysaccharides; MAPK: mitogen-activated protein kinase; mGluR: metabotropic glutamate receptor; MPEP: 2-methyl-6-(phenylethynyl)pyridine hydrochloride; MTEP: 3-((2-methyl-1,3-thiazol-4-yl)ethynyl)pyridine hydrochloride; NFκB: nuclear factor kappa beta; PAMP: pathogen-associated molecular pattern; PBMC: peripheral blood mononuclear cells; PCR: polymerase chain reaction; PHA: phytohemagglutinin; PI3K: phosphatidylinositide 3-kinase; PLC: phospholipase C; TCM: tissue culture medium; TD: typically developing; TLR: Toll-like receptor; TNF: tumor necrosis factor; WT: wild-type.

## Competing interests

RJH has received funding from Novartis, Roche, Seaside Therapeutics, Curemark and Forest to carry out treatment studies in fragile X syndrome and autism. RJH is also on the fragile X advisory boards for Novartis and Roche/Genentech regarding treatment studies. FT has received funding from Roche and consult with Novartis and Genentech. The other authors declare that they have no competing interests.

## Authors’ contributions

MC and PA designed the study. MC and TN performed the experiments in the study. MC and KB analyzed the data performed statistics. FT provided genotyping data. MC, PA, FT, TN, JVW, RJH and KB all took part in writing the manuscript and critically revised the manuscript. All authors read and approved the final manuscript.

## References

[B1] Fu YH Kuhl DPA Pizzuti A Pieretti M Sutcliffe JS Richards S Verkerk AJMH Holden JJA Fenwick RG Warren ST Oostra BA Nelson DL Caskey CT Variation of the Cgg Repeat at the Fragile-X Site Results in Genetic Instability - Resolution of the Sherman Paradox Cell 1991 67 1047 1058 1760838 10.1016/0092-8674(91)90283-5

[B2] Verkerk AJ Pieretti M Sutcliffe JS Fu YH Kuhl DP Pizzuti A Reiner O Richards S Victoria MF Zhang FP Zhang F Eussen BE van Ommen GB Blonden LA Riggins GJ Chastain JL Kunst CB Galjaard H Caskey CT Nelson DL Oostra BA Warren ST Identification of a gene (FMR-1) containing a CGG repeat coincident with a breakpoint cluster region exhibiting length variation in fragile X syndrome Cell 1991 65 905 914 1710175 10.1016/0092-8674(91)90397-h

[B3] Yu S Pritchard M Kremer E Lynch M Nancarrow J Baker E Holman K Mulley JC Warren ST Schlessinger D Sutherland GR Richards RI Fragile X genotype characterized by an unstable region of DNA Science 1991 252 1179 1181 2031189 10.1126/science.252.5009.1179

[B4] Snow K Doud LK Hagerman R Pergolizzi RG Erster SH Thibodeau SN Analysis of a CGG sequence at the FMR-1 locus in fragile X families and in the general population Am J Hum Genet 1993 53 1217 1228 7902673 PMC1682501

[B5] Harris SW Hessl D Goodlin-Jones B Ferranti J Bacalman S Barbato I Tassone F Hagerman PJ Herman H Hagerman RJ Autism profiles of males with fragile X syndrome Am J Ment Retard 2008 113 427 438 19127654 10.1352/2008.113:427-438 PMC2629645

[B6] Hagerman RJ Hall DA Coffey S Leehey M Bourgeois J Gould J Zhang L Seritan A Berry-Kravis E Olichney J Miller JW Fong AL Carpenter R Bodine C Gane LW Rainin E Hagerman H Hagerman PJ Treatment of fragile X-associated tremor ataxia syndrome (FXTAS) and related neurological problems Clin Interv Aging 2008 3 251 262 18686748 10.2147/cia.s1794 PMC2546470

[B7] Ashwood P Nguyen DV Hessl D Hagerman RJ Tassone F Plasma cytokine profiles in fragile X subjects: is there a role for cytokines in the pathogenesis? Brain Behav Immun 2010 24 898 902 20102735 10.1016/j.bbi.2010.01.008 PMC3626458

[B8] Hagerman RJ Altshul-Stark D McBogg P Recurrent otitis media in the fragile X syndrome Am J Dis Child 1987 141 184 187 3812386 10.1001/archpedi.1987.04460020074031

[B9] Bear MF Huber KM Warren ST The mGluR theory of fragile X mental retardation Trends Neurosci 2004 27 370 377 15219735 10.1016/j.tins.2004.04.009

[B10] Ferraguti F Shigemoto R Metabotropic glutamate receptors Cell Tissue Res 2006 326 483 504 16847639 10.1007/s00441-006-0266-5

[B11] Lüscher C Huber KM Group 1 mGluR-dependent synaptic long-term depression: mechanisms and implications for circuitry and disease Neuron 2010 65 445 459 20188650 10.1016/j.neuron.2010.01.016 PMC2841961

[B12] Aronica E Gorter JA Rozemuller AJ Yankaya B Troost D Activation of metabotropic glutamate receptor 3 enhances interleukin (IL)-1beta-stimulated release of IL-6 in cultured human astrocytes Neuroscience 2005 130 927 933 15652990 10.1016/j.neuroscience.2004.10.024

[B13] Boldyrev AA Carpenter DO Johnson P Emerging evidence for a similar role of glutamate receptors in the nervous and immune systems J Neurochem 2005 95 913 918 16271044 10.1111/j.1471-4159.2005.03456.x

[B14] Lombardi G Miglio G Dianzani C Mesturini R Varsaldi F Chiocchetti A Dianzani U Fantozzi R Glutamate modulation of human lymphocyte growth: *in vitro* studies Biochem Biophys Res Commun 2004 318 496 502 15120628 10.1016/j.bbrc.2004.04.053

[B15] Pacheco R Gallart T Lluis C Franco R Role of glutamate on T-cell mediated immunity J Neuroimmunol 2007 185 9 19 17303252 10.1016/j.jneuroim.2007.01.003

[B16] Frank MP Powers RW Simple and rapid quantitative high-performance liquid chromatographic analysis of plasma amino acids J Chromatogr B Analyt Technol Biomed Life Sci 2007 852 646 649 10.1016/j.jchromb.2007.01.002 PMC2201986 17254851

[B17] Clements JD Lester RA Tong G Jahr CE Westbrook GL The time course of glutamate in the synaptic cleft Science 1992 258 1498 1501 1359647 10.1126/science.1359647

[B18] Pacheco R Ciruela F Casado V Mallol J Gallart T Lluis C Franco R Group I metabotropic glutamate receptors mediate a dual role of glutamate in T cell activation J Biol Chem 2004 279 33352 33358 15184389 10.1074/jbc.M401761200

[B19] Geurts JJ Wolswijk G Bo L Redeker S Ramkema M Troost D Aronica E Expression patterns of group III metabotropic glutamate receptors mGluR4 and mGluR8 in multiple sclerosis lesions J Neuroimmunol 2005 158 182 190 15589052 10.1016/j.jneuroim.2004.08.012

[B20] Wechsler D Wechsler Intelligence Scale for Children - Fourth Edition (WISC-IV) 2003 San Antonio: Psychological Corporation, Harcourt Brace

[B21] Sparrow SCD Balla D Vineland Adaptive Behavior Scales, Second Edition (VABS-II): Interview Edition Survey Form 2005 Circle, MN: American Guidance Service

[B22] Rutter MBA Berument SK Lord C Pickles A Social Communication Questionnaire (SCQ) 2003 Western Psychological Services: Los Angeles

[B23] Tassone F Pan R Amiri K Taylor AK Hagerman PJ A rapid polymerase chain reaction-based screening method for identification of all expanded alleles of the fragile X (FMR1) gene in newborn and high-risk populations J Mol Diagn 2008 10 43 49 18165273 10.2353/jmoldx.2008.070073 PMC2175542

[B24] Filipovic-Sadic S Sah S Chen L Krosting J Sekinger E Zhang W Hagerman PJ Stenzel TT Hadd AG Latham GJ Tassone F A novel FMR1 PCR method for the routine detection of low abundance expanded alleles and full mutations in fragile X syndrome Clin Chem 2010 56 399 408 20056738 10.1373/clinchem.2009.136101 PMC4031651

[B25] Vogelzangs N Beekman AT de Jonge P Penninx BW Anxiety disorders and inflammation in a large adult cohort Transl Psychiatry 2013 3 e249 23612048 10.1038/tp.2013.27 PMC3641413

[B26] Dolen G Bear MF Role for metabotropic glutamate receptor 5 (mGluR5) in the pathogenesis of fragile X syndrome J Physiol 2008 586 1503 1508 18202092 10.1113/jphysiol.2008.150722 PMC2375688

[B27] Dolen G Osterweil E Rao BS Smith GB Auerbach BD Chattarji S Bear MF Correction of fragile X syndrome in mice Neuron 2007 56 955 962 18093519 10.1016/j.neuron.2007.12.001 PMC2199268

[B28] Volpi C Fazio F Fallarino F Targeting metabotropic glutamate receptors in neuroimmune communication Neuropharmacology 2012 63 501 506 22640632 10.1016/j.neuropharm.2012.05.024

[B29] McMullan SM Phanavanh B Li GG Barger SW Metabotropic glutamate receptors inhibit microglial glutamate release ASN Neuro 2012 4 323 330 10.1042/AN20120044 PMC3413012 22770428

[B30] Byrnes KR Stoica B Loane DJ Riccio A Davis MI Faden AI Metabotropic glutamate receptor 5 activation inhibits microglial associated inflammation and neurotoxicity Glia 2009 57 550 560 18816644 10.1002/glia.20783 PMC2644739

[B31] Eyo UB Wu L-J Bidirectional microglia-neuron communication in the healthy brain Neural Plasticity 2013 2013 1 10 10.1155/2013/456857 PMC3775394 24078884

[B32] Bilousova TV Dansie L Ngo M Aye J Charles JR Ethell DW Ethell IM Minocycline promotes dendritic spine maturation and improves behavioural performance in the fragile X mouse model J Med Genet 2009 46 94 102 18835858 10.1136/jmg.2008.061796

[B33] Yuskaitis CJ Beurel E Jope RS Evidence of reactive astrocytes but not peripheral immune system activation in a mouse model of fragile X syndrome Biochimica et Biophysica Acta (BBA)-Molecular Basis of Disease 2010 1802 1006 1012 10.1016/j.bbadis.2010.06.015 PMC2942952 20600866

[B34] Sharma A Hoeffer CA Takayasu Y Miyawaki T McBride SM Klann E Zukin RS Dysregulation of mTOR signaling in fragile X syndrome J Neurosci 2010 30 694 702 20071534 10.1523/JNEUROSCI.3696-09.2010 PMC3665010

[B35] Weng N Weiler IJ Sumis A Berry-Kravis E Greenough WT Early-phase ERK activation as a biomarker for metabolic status in fragile X syndrome Am J Med Genet B Neuropsychiatr Genet 2008 147B 1253 1257 18452182 10.1002/ajmg.b.30765

[B36] Juntilla MM Koretzky GA Critical roles of the PI3K/Akt signaling pathway in T cell development Immunol Lett 2008 116 104 110 18243340 10.1016/j.imlet.2007.12.008 PMC2322870

[B37] Yan QJ Rammal M Tranfaglia M Bauchwitz RP Suppression of two major fragile X syndrome mouse model phenotypes by the mGluR5 antagonist MPEP Neuropharmacology 2005 49 1053 1066 16054174 10.1016/j.neuropharm.2005.06.004

[B38] de Vrij FM Levenga J van der Linde HC Koekkoek SK De Zeeuw CI Nelson DL Oostra BA Willemsen R Rescue of behavioral phenotype and neuronal protrusion morphology in Fmr1 KO mice Neurobiol Dis 2008 31 127 132 18571098 10.1016/j.nbd.2008.04.002 PMC2481236

[B39] Aschrafi A Cunningham BA Edelman GM Vanderklish PW The fragile X mental retardation protein and group I metabotropic glutamate receptors regulate levels of mRNA granules in brain Proc Natl Acad Sci U S A 2005 102 2180 2185 15684045 10.1073/pnas.0409803102 PMC548595

[B40] Osterweil EK Krueger DD Reinhold K Bear MF Hypersensitivity to mGluR5 and ERK1/2 leads to excessive protein synthesis in the hippocampus of a mouse model of fragile X syndrome J Neurosci 2010 30 15616 15627 21084617 10.1523/JNEUROSCI.3888-10.2010 PMC3400430

[B41] Lindemann L Jaeschke G Michalon A Vieira E Honer M Spooren W Porter R Hartung T Kolczewski S Buttelmann B Flament C Diener C Fischer C Gatti S Prinssen EP Parrott N Hoffmann G Wettstein JG CTEP: a novel, potent, long-acting, and orally bioavailable metabotropic glutamate receptor 5 inhibitor J Pharmacol Exp Ther 2011 339 474 486 21849627 10.1124/jpet.111.185660

[B42] Berry-Kravis E Huttenlocher PR Cyclic AMP metabolism in fragile X syndrome Ann Neurol 1992 31 22 26 1371909 10.1002/ana.410310105

[B43] Berry-Kravis E Ciurlionis R Overexpression of fragile X gene (FMR-1) transcripts increases cAMP production in neural cells J Neurosci Res 1998 51 41 48 9452307 10.1002/(SICI)1097-4547(19980101)51:1<41::AID-JNR4>3.0.CO;2-L

[B44] Berry-Kravis E Hicar M Ciurlionis R Reduced cyclic AMP production in fragile X syndrome: cytogenetic and molecular correlations Pediatr Res 1995 38 638 643 8552427 10.1203/00006450-199511000-00002

